# Adaptive design for identifying maximum tolerated dose early to accelerate dose-finding trial

**DOI:** 10.1186/s12874-022-01584-y

**Published:** 2022-04-06

**Authors:** Masahiro Kojima

**Affiliations:** 1 Biometrics Department, Kyowa Kirin Co., Ltd, R&D Division, Tokyo, Japan; 2 Department of Statistical Science, School of Multidisciplinary Sciences, The Graduate University for Advanced Studies, Tokyo, Japan

**Keywords:** Time-to-event model-assisted design, Dose-finding design, Early identification of maximum tolerated dose

## Abstract

**Purpose:**

The early identification of maximum tolerated dose (MTD) in phase I trial leads to faster progression to a phase II trial or an expansion cohort to confirm efficacy.

**Methods:**

We propose a novel adaptive design for identifying MTD early to accelerate dose-finding trials. The early identification of MTD is determined adaptively by dose-retainment probability using a trial data via Bayesian analysis. We applied the early identification design to an actual trial. A simulation study evaluates the performance of the early identification design.

**Results:**

In the actual study, we confirmed the MTD could be early identified and the study period was shortened. In the simulation study, the percentage of the correct MTD selection in the early identification Keyboard and early identification Bayesian optimal interval (BOIN) designs was almost same from the non-early identification version. The early identification Keyboard and BOIN designs reduced the study duration by about 50% from the model-assisted designs. In addition, the early identification Keyboard and BOIN designs reduced the study duration by about 20% from time-to-event model-assisted designs.

**Conclusion:**

We proposed the early identification of MTD maintaining the accuracy to be able to short the study period.

**Supplementary Information:**

The online version contains supplementary material available at 10.1186/s12874-022-01584-y.

## Introduction

In the field of oncology, the objective of phase I dose-finding trials is to identify the maximum tolerated dose (MTD). To achieve this goal, model-assisted designs, which combine the simplicity of a 3 + 3 design with the superior performance of a continual reassessment method (CRM) [1], have recently been proposed. Recent studies use the model-assisted designs (Clinical-Trials.gov identifier: NCT04926285, NCT04573140, NCT04390737, NCT05024305, NCT04678921, NCT04511039). However, model-assisted designs cannot proceed to the next cohort until the safety assessment completes because the dose for the next cohort cannot be determined. Hence, rapid patient enrollment or late-onset of toxicity would slow down the study. Therefore, Yuan et al. [2] and Lin et al. [3] proposed a time-to-event (TITE) model-assisted design which determines the dose for the next cohort and proceeds to the next cohort during the safety assessment for the current cohort. However, the study cannot identify the MTD until the number of patients treated reaches the sample size. Early identification methods for MTD have been proposed [4, 5]. However, the methods cannot apply to TITE model-assisted designs.

In this paper, we propose a novel early identification TITE model-assisted designs. The early identification allows to proceed to an expansion cohort or a phase II trial quicker to confirm efficacy. The early identification is determined when the MTD is estimated with sufficient accuracy based on the dose-retainment probability. The early identification method performs on an actual trial. A simulation study evaluates the performance of the early identification method.

## Methods

The TITE model-assisted designs include the modified toxicity probability (mTPI) [6], Keyboard [7], and Bayesian optimal interval (BOIN) [8, 9] designs. A feature of these designs is to provide the number of dose-limiting toxicities (DLTs) to determine the dose assignment as shown in Table 1 [6–8] in advance. For Table 1, we show an example calculation of the BOIN design. For example, the BOIN design [8] conducts a dose-assignment based on a dose-retainment interval (0.236, 0.358) for a target DLT level 30%. The dose-retainment interval is derived to minimize the probability of mis-determination of dose assignment. 0.236 is the maximum boundary for dose escalation and 0.358 is the minimum boundary for dose de-escalation. We derive the number of DLTs of dose escalation and de-escalation for the BOIN design on Table 1 when six patients have been treated. If one patient has occurred a DLT, then the observed DLT rate is 0.167. Because the observed DLT rate is below the maximum boundary for dose escalation, the dose for the next cohort is escalated. If two patients have occurred DLTs, then the observed DLT rate is 0.333. Because the observed DLT rate is within between the maximum boundary for dose escalation and the minimum boundary for dose de-escalation, the dose for the next cohort is retained. The maximum number of DLTs for which a dose escalation is determined is one. Hence, the dose escalation in Table 1 is written as 1. If three patients have occurred DLTs, then the observed DLT rate is 0.500. Because the observed DLT rate is over the minimum boundary for dose de-escalation, the dose for the next cohort is de-escalated. The minimum number of DLTs for which a dose de-escalation is determined is three. Hence, the dose de-escalation in Table 1 is written as 3. It can be calculated in the same way for other numbers of patients treated. In addition, the mTPI and Keyboard design can also be calculated in the same way. The performance of dose assignment using Table 1 is confirmed superior than 3 + 3 design [6–8]. The dose-retainment probability for the early identification is calculated using Table 1. We introduce the dose-retainment probabilities. We assume a phase I dose-finding trial with sample size $N$. The total number of patients treated at the current dose is $n$, the total number of DLTs at the current dose is ${n}_{DLT}$, the total number of no DLTs patients who have completed the safety assessment at the current dose is ${n}_{noDLT}$, the no DLT time of pending patients at the current dose is ${t}_{pend}$, the DLT assessment window is $t$, the no DLT time rate is ${n}_{pend}=\frac{{t}_{pend}}{t}$, the estimated number of no DLT patients is ${n}_{e}={n}_{noDLT}+{n}_{pend}$, the number of remaining patients is $r$, the number of DLTs of dose escalation decision at $n+r$ patients in the dose-assignment table is ${E}_{n+r}$, the number of DLTs of dose de-escalation decision at $n+r$ patients in the dose-assignment table is ${D}_{n+r}$. A value ${r}_{pend}$ is the number of remaining patients $r$ plus the no DLT time rate ${n}_{pend}$.

**Table 1:** Dose escalation and de-escalation boundaries (TTL = 0.3)

Design	Action	Num of patients treated at current dose					
		3	6	9	12	15	18
mTPI	Escalate if Num of DLTs $\le$	0	1	1	2	2	3
	De-escalate if Num of DLTs $\ge$	2	3	4	5	7	8
Keyboard	Escalate if Num of DLTs $\le$	0	1	2	2	3	4
	De-escalate if Num of DLTs $\ge$	2	3	4	5	6	7
BOIN	Escalate if Num of DLTs $\le$	0	1	2	2	3	4
	De-escalate if Num of DLTs $\ge$	2	3	4	5	6	7

The dose-retainment probability is given by

$$BB\left({D}_{n+r}-1-{n}_{DLT};{r}_{pend},{n}_{DLT},{n}_{DLT}+{n}_{e}\right)-BB\left({E}_{n+r}-{n}_{DLT};{r}_{pend},{n}_{DLT},{{n}_{DLT}+n}_{e}\right).$$

$BB\left(a;b,\alpha ,\beta \right)$ is the cumulative beta-binomial distribution function with the number of successes $a$, the number of trials $b$, and the beta shape parameter $\alpha$ and $\beta$. $BB\left({D}_{n+r}-1-{n}_{DLT};{r}_{pend},{n}_{DLT},{n}_{DLT}+{n}_{e}\right)$ refers to the dose not de-escalation probability for ${r}_{pend}$ patients using the maximum value for dose not de-escalation (${D}_{n+r}-1-{n}_{DLT}$). Because, the probability includes the dose escalation probability, we take the difference with the dose escalation probability $BB\left({E}_{n+r}-{n}_{DLT};{r}_{pend},{n}_{DLT},{n}_{DLT}+{n}_{e}\right)$. The threshold for early identification of MTD is $t$. If the probability of dose maintenance exceeds $t$, the trial can halt and the MTD is identified. The recommended value of the threshold is $0.4$. The rationale of the recommended value is explained by Kojima [5]. At the maximum dose, because there is no dose escalation, the early identification is determined by $BB\left({D}_{n+r}-1-{n}_{DLT};{r}_{pend},{n}_{DLT},{n}_{DLT}+{n}_{e}\right)$. At minimum dose, because there is no dose de-escalation, the early identification is determined by $1-BB\left({E}_{n+r}-{n}_{DLT};{r}_{pend},{n}_{DLT},{n}_{DLT}+{n}_{e}\right)$. The threshold value at the maximum and minimum doses is twice. Hence, the recommended value is 0.8. If there is no DLT at the current dose, the conditional ${n}_{DLT}$ and $n$ of the cumulative beta-binomial distribution function are added 0.5. The rationale of adding 0.5 is explained by Kojima [5].

**Fig. 1:**
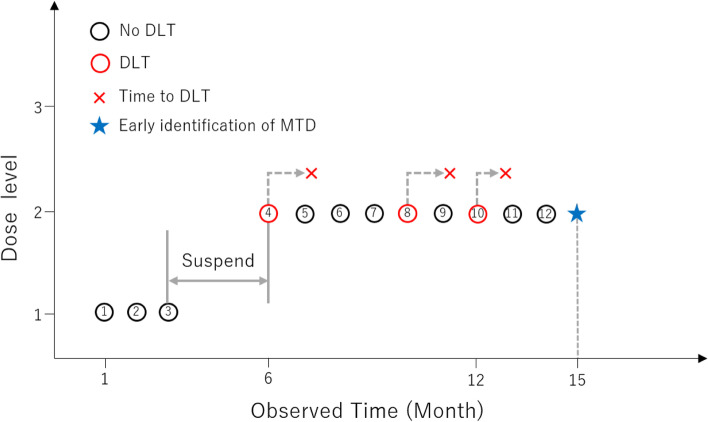
Example

We present a numerical example for an early identification TITE-BOIN design with a target DLT level 30%. The DLT assessment window is three months. The patient enrollment is one patient per month. The sample size is $N=18$, the total patients treated is $12$, $n=9$ patients received the current dose, the total number of DLTs at the current dose is ${n}_{DLT}=3$, the total number of no DLTs patients evaluated at the current dose is ${n}_{noDLT}=4$, two patients are pending at the current dose. We illustrate this example in Fig. 1. The pending time for the eleventh patient is two months and the pending time for the twelfth patient is one month. The no DLT time of pending patient at the current dose is ${t}_{p}=3$, the total evaluation time of pending patient at the current dose is $t=3$, the no DLT time rate is ${n}_{p}=\frac{{t}_{p}}{t}=\frac{3}{3}=1.0$, the estimated number of no DLT patients is ${n}_{e}={n}_{noDLT}+{n}_{p}=4+1=5$, the number of remaining patients including no DLT time is $r=6$. From Table 1, ${E}_{n+r}={E}_{15}=3$ and ${D}_{n+r}={D}_{15}=6$. The dose not de-escalation probability is $BB\left(3-1;6+\mathrm{1.0,3},5\right)=0.500$ and dose-escalation probability is $BB\left(1-1;6+\mathrm{1,3},5\right)=0.096$. Hence, the dose retainment probability is $0.404$, and the probability is above the threshold 0.4. Therefore, the early identification of MTD is determined and we can halt the MTD estimation phase. If the sample size is $N=21$ and the number of remaining patients including no DLT time is $r=9$, the dose not de-escalation probability is $BB\left(3-1;9+\mathrm{1.0,3},5\right)=0.404$ and dose-escalation probability is $BB\left(1-1;9+\mathrm{1,3},5\right)=0.203$. Hence, the dose retainment probability is $0.141$. In this case, we cannot halt the MTD estimation phase early. Because the number of remaining patients is large, the dose retainment probability decreases due to increased uncertainty.

We apply the early identification to an actual trial. The TITE-model assisted designs are new designs and there are no completed studies. Hence, the early identification performs for an actual study with TITE continual reassessment method (CRM) design with similar performance to the TITE model-assisted designs.

### TBCRC 024 trial as an illustrative example

The TBCRC 024 trial [10] was a phase I trial using the time-to-event CRM (TITE-CRM) design for the chest wall and regional lymph nodes in patients with inflammatory or locally recurrent breast cancer after complete surgical resection. The primary objective was to determine the MTD of veliparib in combination with chest wall and nodal radiotherapy. The safety assessment period is 10 weeks (a 70-day time period). The planned four dosages of veliparib were 50 mg, 100 mg, 150 mg, and 200 mg, which were taken orally twice a day. The sample size was 30. The target DLT level was 30%. The cohort size was three. The number of patients treated and DLTs at each dose were 50 mg ($n=3$, ${n}_{DLT}=0$), 100 mg ($n=6$, ${n}_{DLT}=2$), 150 mg ($n=12$, ${n}_{DLT}=2$), and 200 mg ($n=9$, ${n}_{DLT}=1$). Although we cannot confirm the DLT status for each cohort from the paper, we assume that the dose was not been reduced after administration of 200 mg because the DLT was only observed once at 200 mg. We consider whether the trial can be completed early after the initial administration of 200 mg starts. We assume that the enrollment is one patient per 60-day.

[No DLT in the first cohort] We assume that the safety evaluation of two patients treated completed and the third patient with no DLT has been observed for up to 35 days. The dose-retainment probabilities of the TITE-mTPI, TITE-Keyboard, and TITE-BOIN are 0.93. The probability is over the threshold 0.8. Hence, we identify the MTD. By the early identification, the study period was shortened by 395 days (395 days = 25 days (the third patient’s remaining safety assessment duration) + 5 $\times$ 60 days (the remaining five patients’ safety assessment duration) + 70 days (the last patient’s safety assessment duration)).

[One DLT in the first cohort] We assume that the safety evaluation of two patients treated completed and one patient occurs a DLT. The third patient with no DLT has been observed for up to 35 days. The all dose-retainment probabilities of the three TITE model-assisted designs are 0.55. We cannot identify the MTD early. For the second cohort, the safety evaluation of two patients treated completed and the third patient with no DLT has been observed for up to 35 days. The dose-retainment probabilities of the TITE-mTPI, TITE-Keyboard, and TITE-BOIN are 0.98. The probability is over the threshold 0.8. Hence, we identify the MTD. By the early identification, the study period was shortened by 215 days. (215 days = 25 days (the sixth patient’s remaining safety assessment duration) + 2 $\times$ 60 days (the remaining two patients’ safety assessment duration) + 70 days (the last patient’s safety assessment duration)).

We evaluate the performance of the early identification of MTD via a simulation study.

### Numerical simulation study

We demonstrate a simulation study to compare early identification TITE mTPI (EI-TITE-mTPI), early identification TITE Keyboard (EI-TITE-Keyboard), and early identification TITE BOIN (EI-TITE-BOIN) designs with mTPI, TITE mTPI, Keyboard, TITE Keyboard, BOIN, and TITE BOIN designs. We imitated simulation setup by Lin et al. [3]. We assume that the sample size is 36, the dose level is six. The DLT assessment window is three months. The patient enrollment is two patients per month. The target DLT level is 30%. The number of simulations times is 10,000. The threshold for early identification of MTD is 0.4. For the mTPI and Keyboard designs, the proper dosing interval is $\left(\mathrm{0.25,0.35}\right)$. For the BOIN design, the dose retainment interval is $\left(0.236, 0.358\right)$. We prepare a fixed scenario and a randomly set scenario for the true DLT rate of each dose. We prohibit the dose skipping for all designs. To avoid assigning many patients treated to the overly DLT dose, we apply the dose elimination rule [2] which excludes the over dosing in the dose-finding trial. We evaluated each method using the following criteria.

### Evaluation criteria

1. The percentage of correct MTD selection (PCMS)
2. The percentage of early identification of MTD
3. Percent change from non-EI version in average study duration
4. Percent change from non-EI version in average sample size

## Results

### Performance for the selection of the correct MTD

**Fig. 2:**
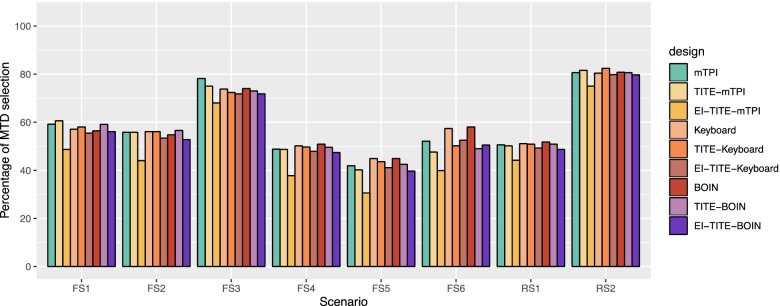
Percentage of MTD selection. FS1: Fixed Scenario 1; FS2: Fixed Scenario 2; FS3: Fixed Scenario 3; FS4: Fixed Scenario 4; FS5: Fixed Scenario 5; FS6: Fixed Scenario 6; RS1: Random Scenario 1; and RS2: Random Scenario 2. EI: Early identification of MTD

Figure 2 illustrates the percentage of the correct MTD selection (PCMS) for the six fixed scenarios and two random scenarios. The EI-TITE-Keyboard and EI-TITE-BOIN designs have almost the same PCMS as the non-EI version. The EI-TITE-Keyboard design have at most 2.7% lower PCMS in Scenario 2 and most 2.3% higher PCMS in Scenario 5 compared to the TITE-Keyboard design. The EI-TITE-BOIN design have at most 3.8% lower PCMS in Scenario 2 and most 1.5% higher PCMS in Scenario 5 compared to the TITE-Keyboard design. The PCMSs of EI-TITE-mTPI design are lower than the non-EI versions, most 12.0% lower in scenario 2. We showed the detail results of each scenario in Supplemental Table 3 and 4.

### Percentage of early identification of MTD

**Fig. 3:**
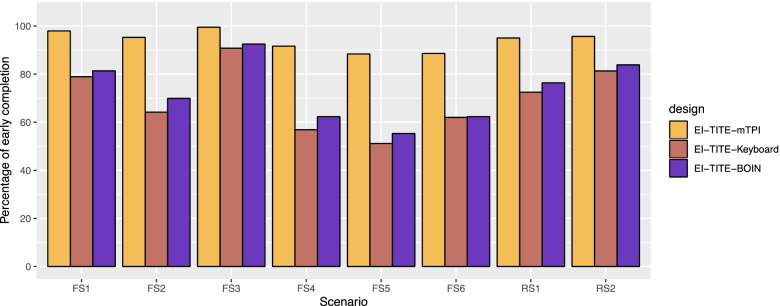
Percentage of early identification of MTD. FS1: Fixed Scenario 1; FS2: Fixed Scenario 2; FS3: Fixed Scenario 3; FS4: Fixed Scenario 4; FS5: Fixed Scenario 5; FS6: Fixed Scenario 6; RS1: Random Scenario 1; and RS2: Random Scenario 2. EI: Early identification of MTD

Figure 3 illustrates the percentage of early identification of MTD. The percentages of early identification for EI-TITE-mTPI range from 88.4% to 98.0%, with the average of 94.0% for all scenarios. The percentages of early identification for EI-TITE-Keyboard range from 51.2% to 90.8%, with the average of 69.7% for all scenarios. The percentages of early identification for EI-TITE-BOIN range from 55.3% to 92.5%, with the average of 73.0% for all scenarios. The EI-TITE-mTPI has the highest percentage of early identification. We confirmed that the EI-TITE-Keyboard and EI-TITE-BOIN designs are able to identify early about 70% on average.

### Percent change from non-EI version in average study duration

**Fig. 4:**
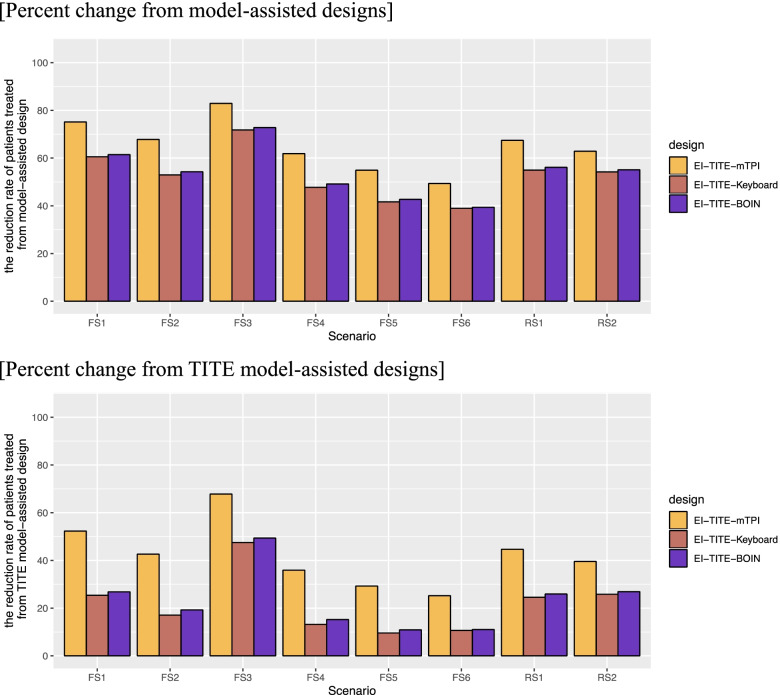
Percent change non-EI version to EI version in average study duration. FS1: Fixed Scenario 1; FS2: Fixed Scenario 2; FS3: Fixed Scenario 3; FS4: Fixed Scenario 4; FS5: Fixed Scenario 5; FS6: Fixed Scenario 6; RS1: Random Scenario 1; and RS2: Random Scenario 2. EI: Early identification of MTD

Figure 4 illustrates two bar charts of the percent change from model-assisted designs and TITE model-assisted designs to EI-TITE model-assisted designs in average study duration. For the percent change from the model-assisted designs, the EI-TITE-mTPI design reduces the study duration by 49.4% to 82.9%, with an average reduction of 65.3%. Thus, the study duration is reduced by 31.3 months on average. The EI-TITE-Keyboard design reduces the study duration by 39.0% to 71.8%, with an average reduction of 52.9%. Thus, the study duration is reduced by 25.4 months on average. The EI-TITE-BOIN design reduces the study duration by 39.4% to 72.8%, with an average reduction of 53.9%. Thus, the study duration is reduced by 25.9 months on average. For the percent change from the TITE model-assisted designs, the EI-TITE-mTPI design reduces the study duration by 25.2% to 67.8%, with an average reduction of 42.2%. Thus, the study duration is reduced by 11.7 months on average. The EI-TITE-Keyboard design reduces the study duration by 9.6% to 47.5%, with an average reduction of 21.7%. Thus, the study duration is reduced by 6.0 months on average. The EI-TITE-BOIN design reduces the study duration by 10.9% to 49.4%, with an average reduction of 23.2%. Thus, the study duration is reduced by 6.4 months on average. We show the summary of percent change from model-assisted designs and TITE model-assisted designs to EI-TITE model-assisted designs in average study duration in Supplemental Table 5. We show the average observed study duration in Supplemental Fig. 1.

### Percent change from non-EI version in average sample size

**Fig. 5:**
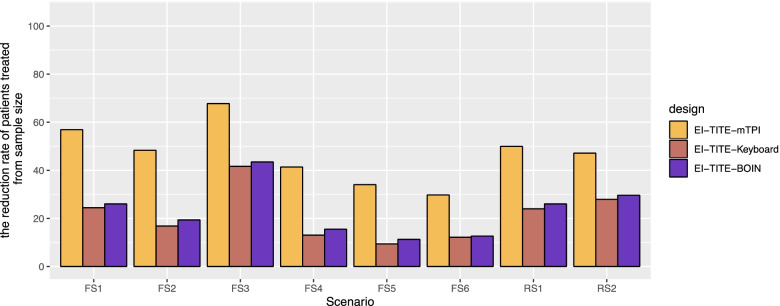
Percent change from planned sample size in EI version. FS1: Fixed Scenario 1; FS2: Fixed Scenario 2; FS3: Fixed Scenario 3; FS4: Fixed Scenario 4; FS5: Fixed Scenario 5; FS6: Fixed Scenario 6; RS1: Random Scenario 1; and RS2: Random Scenario 2. EI: Early identification of MTD

Figure 5 illustrates the percent change from non-EI versions to EI versions in average sample size. The EI-TITE-mTPI design reduces the number of patients treated by 29.8% to 67.7%, with an average reduction of 46.9%. The EI-TITE-Keyboard design reduces the number of patients treated by 9.4% to 41.6%, with an average reduction of 21.2%. The EI-TITE-BOIN design reduces the number of patients treated by 11.3% to 43.5%, with an average reduction of 23.0%.

## Discussion

We proposed a novel adaptive design for identifying MTD early to accelerate dose-finding trials. The early identification is determined adaptively depending on the toxicity data of the trial. The early identification of MTD leads to faster progression to a phase II trial and expansion cohorts to confirm efficacy. We confirmed that the design adapting early identification does not degrade accuracy compared to conventional designs.

We applied the early identification design to an actual trial (TBCRC 024). The MTD were identified early, and we confirmed that the trial could be shortened by about six months to a year.

The simulation study evaluated the performance of the early identification design. We confirmed that the percentage of the correct MTD selection (PCMS) in the early identification Keyboard and early identification BOIN designs was almost same from the non-early identification version. We found that the early identification mTPI design reduced the PCMS from the non-early identification version by about 10% mTPI. The average percentage of early identification was 94.0% for mTPI and approximately 70% for Keyboard and BOIN designs. The mTPI design had a higher probability of dose maintenance than the other designs because the number of DLTs for which dose maintenance was judged was larger, and thus the early completion rate was higher. On the other hand, the PCMS of mTPI design was low because the early identification was determined even in cases that should not have been determined as early identification. We showed the PCMS of for EI designs only when the MTD is identified early in Supplemental Fig. 2 and Supplemental Table 8. For the fixed scenarios 1–4, the PCMSs are almost same compared the EI keyboard and BOIN designs with non-EI keyboard and BOIN designs. For the fixed scenario 5, the PCMSs for EI designs decrease compared to non-EI designs. We considered that the close DLT rate of the correct MTD and nearby doses reduced the accuracy of the early identification. When the DLT rate between doses is assumed to be close in advance, we can change the threshold for early identification to increase the PCMS. For example, when we change a threshold value of 0.5 for scenario 5 from a threshold value of 0.4, the PCMS of EI-keyboard design was improved to 41.2% from 32.3% and the PCMS of EI-BOIN design was improved to 35.9% from 30.6%. For the fixed scenario 6, the PCMSs for EI-TITE-Keyboard and EI-TITE-BOIN are higher than the non-EI designs. The random scenarios 1 and 2, the PCMSs for EI-TITE-Keyboard and EI-TITE-BOIN are similar to the non-EI designs. The early identification Keyboard and BOIN designs reduced the study duration by about 50% from the model-assisted designs. A 50% reduction in the simulation refers to a reduction of about two years. In addition, the early identification Keyboard and BOIN designs reduced the study duration by about 20% from the TITE model-assisted designs. A 20% reduction in the simulation refers to a reduction of about half year. The early identification Keyboard and BOIN designs reduced the number of cases by about 20% from the non-early identification version. Shortening the study duration and reducing the number of patients treated allow for more efficient drug development, as patients who were scheduled to be treated in the MTD estimation phase can be enrolled in phase II trials or expanded cohorts earlier.

We confirmed that the performance of the early identification Keyboard and BOIN designs is better. There is little difference in performance between the early identification keyboard and BOIN designs, but we recommend the keyboard design because it has slightly better performance.

## Supplementary Information

**Additional file 1. **Supplemental data.

## Data Availability

We have used data published in Jagsi et al. (2018).
