# Supplementary material for: Adaptive design for identifying maximum tolerated dose early to accelerate dose-finding trial
Source: BMC Med Res Methodol. 2022 Apr 6;22:97. doi: 10.1186/s12874-022-01584-y (PMC8985324; doi:10.1186/s12874-022-01584-y)
Supplement: Supplementary file 1 — Additional file 1. Supplemental data. [file 12874_2022_1584_MOESM1_ESM.pdf]

Supplemental data

- **Supplemental Table 1.** Fixed DLT probability scenarios
- **Supplemental Table 2.** Algorithm of generating random DLT
- **Supplemental Table 3.** Results of fixed scenarios
- **Supplemental Table 4.** Results of random scenarios
- **Supplemental Table 5.** Percent change in average study duration
- **Supplemental Table 6.** Sensitivity analysis with sample size of 21
- **Supplemental Table 7.** Detailed Dose escalation and de-escalation boundaries (TTL=0.3)
- **Supplemental Table 8.** Percentage of the MTD selection for EI designs only when the MTD is identified early
- **Supplemental Figure 1.** Average observed duration
- **Supplemental Figure 2.** Percentage of the correct MTD selection for EI designs only when the MTD is identified early.

Supplemental Table 1. Fixed DLT probability scenarios

	Dose Level					
	1	2	3	4	5	6

Scenario 1	0.13	**0.28**	0.41	0.50	0.60	0.70
Scenario 2	0.08	0.15	**0.29**	0.43	0.50	0.57
Scenario 3	**0.28**	0.42	0.49	0.61	0.76	0.87
Scenario 4	0.05	0.10	0.20	**0.31**	0.50	0.70
Scenario 5	0.06	0.08	0.12	0.18	**0.30**	0.41
Scenario 6	0.05	0.06	0.08	0.11	0.19	**0.32**

Bold letters are the correct MTD.

Supplemental Table 2. Algorithm of generating random DLT probability

Algorithm 1	The MTD is randomly selected from 6 doses with a uniform probability.
Algorithm 2	The DLT probability of the dose j selected as the MTD is $p_j = \Phi(\epsilon_j), \epsilon_j \sim \mathcal{N}(\Phi^{-1}(0.3), 0.05^2)$, where Φ is the cumulative normal distribution function and Φ^{-1} the inverse of cumulative normal distribution function.
Algorithm 3	The DLT probabilities of the previous and next dose of the MTD is $p_{j-1} = \Phi\left(\Phi^{-1}(p_j) + \left(\Phi^{-1}(0.6 - p_j) - \Phi^{-1}(p_j)\right) I(p_j > 0.3) - \epsilon_{j-1}^2\right), p_{j+1} = \Phi\left(\Phi^{-1}(p_j) + \left(\Phi^{-1}(0.6 - p_j) - \Phi^{-1}(p_j)\right) I(p_j <$

	$0.3) + \epsilon_{j+1}^2\big), \epsilon_{j-1} \sim \mathcal{N}(\mu, 0.05^2), \epsilon_{j+1} \sim \mathcal{N}(\mu, 0.05^2)$.
Algorithm 4	The DLT probabilities at two doses apart from the MTD are $p_{j+k}, p_{j+k}, k \geq 2, p_{j-k} = \Phi\big(\Phi^{-1}(p_{j-k+1}) - \epsilon_{j-k}^2\big), p_{j+k} = \Phi\big(\Phi^{-1}(p_{j+k-1}) - \epsilon_{j+k}^2\big), \epsilon_{j-k} \sim \mathcal{N}(\mu, 0.05^2), \epsilon_{j+k} \sim \mathcal{N}(\mu, 0.05^2)$.

We simulated two scenarios with the value of μ. The scenario 1 is $\mu = 0.5$, the scenario 2 is $\mu = 1.0$.

Supplemental Table 3. Results of fixed scenarios

Design		Dose Level						Ave Pts	%reduc Pts	%early ident	Duration
		1	2	3	4	5	6				
Scenario 1	Pr Tox	0.13	**0.28**	0.41	0.50	0.60	0.70				
mTPI	%MTD	16.1	**59.2**	21.6	3.0	0.2	0.0	36.0			48.0
TITE-mTPI	%MTD	13.5	**60.6**	21.9	3.7	0.3	0.0	36.0			25.0
EI-TITE-mTPI	%MTD	24.5	**48.7**	21.9	4.6	0.4	0.0	15.5	56.9	98.0	11.9
Keyboard	%MTD	14.5	**57.1**	23.9	4.2	0.3	0.0	36.0			48.0
TITE-Keyboard	%MTD	12.8	**58.0**	24.4	4.3	0.5	0.0	36.0			25.4
EI-TITE-Keyboard	%MTD	15.4	**55.5**	24.0	4.6	0.4	0.0	27.2	24.5	78.9	18.9
BOIN	%MTD	15.2	**56.4**	24.2	3.9	0.4	0.0	36.0			48.0
TITE-BOIN	%MTD	13.2	**59.1**	23.2	4.1	0.4	0.0	36.0			25.3
EI-TITE-BOIN	%MTD	15.2	**56.1**	23.9	4.4	0.4	0.0	26.6	26.0	81.4	18.5

Design		Dose Level						Ave Pts	%reduc Pts	%early ident	Duration
		1	2	3	4	5	6				
Scenario 2	Pr Tox	0.08	0.15	**0.29**	0.43	0.50	0.57				
mTPI	%MTD	1.4	22.8	**55.8**	17.2	2.6	0.3	36.0			48.0
TITE-mTPI	%MTD	0.9	21.3	**55.8**	19.0	2.8	0.2	36.0			26.9
EI-TITE-mTPI	%MTD	8.1	26.2	**44.0**	18.4	3.1	0.3	18.6	48.3	95.3	15.4
Keyboard	%MTD	0.9	20.4	**56.1**	18.6	3.6	0.4	36.0			48.0
TITE-Keyboard	%MTD	0.9	20.1	**56.1**	19.0	3.6	0.3	36.0			27.2
EI-TITE-Keyboard	%MTD	1.8	21.1	**53.4**	19.6	3.4	0.6	29.9	16.8	64.2	22.6
BOIN	%MTD	1.0	21.0	**54.8**	19.2	3.7	0.3	36.0			48.0
TITE-BOIN	%MTD	1.0	19.9	**56.6**	19.1	3.2	0.3	36.0			27.2
EI-TITE-BOIN	%MTD	2.0	21.3	**52.8**	20.1	3.2	0.6	29.0	19.4	69.9	22.0
Scenario 3	Pr Tox	**0.28**	0.42	0.49	0.61	0.76	0.87				
mTPI	%MTD	**78.2**	19.1	2.6	0.1	0.0	0.0	36.0			48.0
TITE-mTPI	%MTD	**75.0**	21.9	2.9	0.2	0.0	0.0	36.0			25.5
EI-TITE-mTPI	%MTD	**68.0**	26.5	4.9	0.6	0.0	0.0	11.6	67.7	99.5	8.2
Keyboard	%MTD	**73.8**	22.6	3.4	0.2	0.0	0.0	36.0			48.0
TITE-Keyboard	%MTD	**72.4**	23.5	3.8	0.3	0.0	0.0	36.0			25.8
EI-TITE-Keyboard	%MTD	**71.8**	23.5	4.3	0.4	0.0	0.0	21.0	41.6	90.8	13.5
BOIN	%MTD	**74.0**	22.4	3.4	0.2	0.0	0.0	36.0			48.0
TITE-BOIN	%MTD	**73.0**	22.9	3.8	0.3	0.0	0.0	36.0			25.8

Design		Dose Level						Ave Pts	%reduc Pts	%early ident	Duration
		1	2	3	4	5	6				
EI-TITE-BOIN	%MTD	**71.8**	23.6	4.3	0.3	0.0	0.0	20.3	43.5	92.5	13.0
Scenario 4	Pr Tox	0.05	0.10	0.20	**0.31**	0.50	0.70				
mTPI	%MTD	0.2	5.0	36.7	**48.8**	9.1	0.2	36.0			48.0
TITE-mTPI	%MTD	0.2	4.5	35.7	**48.7**	10.7	0.3	36.0			28.6
EI-TITE-mTPI	%MTD	2.9	12.9	35.1	**37.8**	10.9	0.5	21.1	41.4	91.6	18.3
Keyboard	%MTD	0.2	4.4	32.9	**50.2**	12.0	0.4	36.0			48.0
TITE-Keyboard	%MTD	0.1	3.7	33.9	**49.7**	12.1	0.5	36.0			28.9
EI-TITE-Keyboard	%MTD	0.3	5.1	33.4	**47.9**	12.7	0.6	31.3	13.0	56.9	25.1
BOIN	%MTD	0.2	4.3	32.5	**50.9**	11.9	0.3	36.0			48.0
TITE-BOIN	%MTD	0.1	4.3	34.5	**49.6**	11.3	0.3	36.0			28.8
EI-TITE-BOIN	%MTD	0.3	6.0	33.5	**47.4**	12.3	0.5	30.4	15.5	62.3	24.4
Scenario 5	Pr Tox	0.06	0.08	0.12	0.18	**0.30**	0.41				
mTPI	%MTD	0.2	1.2	7.7	31.4	**41.9**	17.6	36.0			48.0
TITE-mTPI	%MTD	0.1	1.7	9.3	32.1	**40.2**	16.6	36.0			30.6
EI-TITE-mTPI	%MTD	3.8	7.0	14.4	29.2	**30.6**	15.0	23.7	34.0	88.4	21.6
Keyboard	%MTD	0.1	0.6	5.4	27.8	**44.9**	21.2	36.0			48.0
TITE-Keyboard	%MTD	0.1	1.1	7.0	30.5	**43.6**	17.8	36.0			31.0
EI-TITE-Keyboard	%MTD	0.3	1.6	8.5	29.2	**41.1**	19.2	32.6	9.4	51.2	28.0
BOIN	%MTD	0.1	0.8	5.8	27.3	**44.9**	21.1	36.0			48.0

Design		Dose Level						Ave Pts	%reduc Pts	%early ident	Duration
		1	2	3	4	5	6				
TITE-BOIN	%MTD	0.1	1.0	7.7	31.7	**42.5**	17.1	36.0			30.9
EI-TITE-BOIN	%MTD	0.5	1.7	9.0	30.6	**39.7**	18.6	31.9	11.3	55.3	27.5
Scenario 6	Pr Tox	0.05	0.06	0.08	0.11	0.19	**0.32**				
mTPI	%MTD	0.1	0.2	1.5	9.4	36.6	**52.1**	36.0			48.0
TITE-mTPI	%MTD	0.1	0.5	2.7	11.2	37.9	**47.6**	36.0			32.5
EI-TITE-mTPI	%MTD	2.9	4.1	6.8	13.8	32.5	**39.9**	25.3	29.8	88.6	24.3
Keyboard	%MTD	0.0	0.2	1.0	6.7	34.7	**57.4**	36.0			48.0
TITE-Keyboard	%MTD	0.0	0.3	1.5	9.7	38.2	**50.2**	36.0			32.8
EI-TITE-Keyboard	%MTD	0.2	0.6	2.2	9.3	35.2	**52.5**	31.6	12.2	62.0	29.3
BOIN	%MTD	0.0	0.2	0.9	6.4	34.5	**58.0**	36.0			48.0
TITE-BOIN	%MTD	0.0	0.4	2.0	10.5	38.1	**49.0**	36.0			32.7
EI-TITE-BOIN	%MTD	0.2	0.7	2.4	10.6	35.5	**50.5**	31.4	12.6	62.3	29.1

Target DLT level is 30%. Pr Tox: DLT probability, %MTD: the percentage of correct MTD selection, Ave Pts, the average number of patients treated, %reduc Pts: the reduction rate of patients treated from planned sample size, %early indent, the percentage of early identification, Duration: average study duration. Bold letters are the correct MTD.

Supplemental Table 4. Results of random scenarios

Design	%MTD	Ave Pts	%reduc Pts	%early comp	Duration
Random Scenario 1 ($\mu = 0.5$)					

Design	%MTD	Ave Pts	%reduc Pts	%early comp	Duration
mTPI	50.6	36.0			48.0
TITE-mTPI	50.2	36.0			28.3
EI-TITE-mTPI	44.2	18.0	50.0	95.0	15.6
Keyboard	51.1	36.0			48.0
TITE- Keyboard	50.9	36.0			28.6
EI-TITE- Keyboard	49.3	27.4	24.0	72.5	21.6
BOIN	51.8	36.0			48.0
TITE- BOIN	50.9	36.0			28.4
EI-TITE- BOIN	48.7	26.6	26.0	76.4	21.1
Random Scenario 2 ($\mu = 1.0$)					
mTPI	80.6	36.0			48.0
TITE-mTPI	81.6	36.0			29.5
EI-TITE-mTPI	75.0	19.0	47.2	95.7	17.8
Keyboard	80.4	36.0			48.0
TITE- Keyboard	82.4	36.0			29.6
EI-TITE- Keyboard	79.8	25.9	27.9	81.3	22.0
BOIN	80.8	36.0			48.0
TITE- BOIN	80.6	36.0			29.5
EI-TITE- BOIN	79.7	25.3	29.6	83.9	21.5

Target DLT level is 30%. %MTD: the percentage of correct MTD selection, Ave Pts, the average number of patients treated, %reduc Pts: the reduction rate of patients treated from planned sample size, %early indent, the percentage of early identification, Duration: average study duration.

Supplemental Table 5. Percent change in average study duration

Design	Percent change from model-assisted designs in average study duration	Percent change from TITE model-assisted designs in average study duration
Fixed Scenario 1		
EI-TITE-mTPI	75.2	52.3
EI-TITE- Keyboard	60.6	25.4

Design	Percent change from model-assisted designs in average study duration	Percent change from TITE model-assisted designs in average study duration
EI-TITE- BOIN	61.5	26.8
Fixed Scenario 2		
EI-TITE-mTPI	67.8	42.6
EI-TITE- Keyboard	53.0	17.1
EI-TITE- BOIN	54.3	19.2
Fixed Scenario 3		
EI-TITE-mTPI	82.9	67.8
EI-TITE- Keyboard	71.8	47.5
EI-TITE- BOIN	72.8	49.4
Fixed Scenario 4		
EI-TITE-mTPI	61.9	35.9
EI-TITE- Keyboard	47.7	13.2
EI-TITE- BOIN	49.2	15.2
Fixed Scenario 5		
EI-TITE-mTPI	54.9	29.3
EI-TITE- Keyboard	41.7	9.6
EI-TITE- BOIN	42.7	10.9
Fixed Scenario 6		
EI-TITE-mTPI	49.4	25.2
EI-TITE- Keyboard	39.0	10.7
EI-TITE- BOIN	39.4	11.0
Random Scenario 1 ($\mu = 0.5$)		
EI-TITE-mTPI	67.4	44.7
EI-TITE- Keyboard	55.0	24.5
EI-TITE- BOIN	56.1	25.9
Random Scenario 2 ($\mu = 1.0$)		
EI-TITE-mTPI	62.9	39.6
EI-TITE- Keyboard	54.2	25.8
EI-TITE- BOIN	55.1	26.9

Supplemental Table 6. Sensitivity analysis with sample size of 21

Design	%MTD	Ave Pts	%reduc Pts	%early comp	Duration
Fixed Scenario 1					
mTPI	51.3	21.0			28.0
TITE-mTPI	52.0	21.0			17.2
EI-TITE-mTPI	49.8	14.2	32.2	75.1	11.8
Keyboard	50.5	21.0			28.0
TITE- Keyboard	52.2	21.0			17.4
EI-TITE- Keyboard	52.0	19.2	8.6	47.6	15.6
BOIN	51.2	21.0			28.0
TITE- BOIN	52.5	21.0			17.3
EI-TITE- BOIN	51.0	18.8	10.5	49.7	15.3
Fixed Scenario 2					
mTPI	49.3	21.0			28.0
TITE-mTPI	48.4	21.0			18.8
EI-TITE-mTPI	44.7	15.9	24.2	64.4	14.6
Keyboard	48.8	21.0			28.0
TITE- Keyboard	49.9	21.0			19.0
EI-TITE- Keyboard	49.6	19.9	5.4	29.2	17.9
BOIN	49.5	21.0			28.0
TITE- BOIN	49.5	21.0			19.0
EI-TITE- BOIN	48.7	19.7	6.2	31.0	17.7
Fixed Scenario 3					
mTPI	73.3	21.0			28.0
TITE-mTPI	70.3	21.0			16.5
EI-TITE-mTPI	69.6	11.3	46.4	86.7	8.3
Keyboard	69.2	21.0			28.0
TITE- Keyboard	69.5	21.0			16.8
EI-TITE- Keyboard	69.7	16.8	19.8	61.7	12.3
BOIN	69.8	21.0			28.0
TITE- BOIN	69.6	21.0			16.6

Design	%MTD	Ave Pts	%reduc Pts	%early comp	Duration
EI-TITE- BOIN	71.8	15.4	26.8	67.6	11.4
Fixed Scenario 4					
mTPI	38.3	21.0			28.0
TITE-mTPI	33.9	21.0			20.2
EI-TITE-mTPI	28.4	16.8	19.9	58.8	16.5
Keyboard	41.7	21.0			28.0
TITE- Keyboard	36.9	21.0			20.4
EI-TITE- Keyboard	36.9	20.3	3.6	19.6	19.6
BOIN	40.5	21.0			28.0
TITE- BOIN	36.5	21.0			20.3
EI-TITE- BOIN	36.8	20.1	4.1	21.3	19.4
Fixed Scenario 5					
mTPI	30.0	21.0			28.0
TITE-mTPI	26.8	21.0			21.4
EI-TITE-mTPI	23.8	17.6	16.2	53.5	18.3
Keyboard	35.1	21.0			28.0
TITE- Keyboard	27.9	21.0			21.8
EI-TITE- Keyboard	28.3	20.6	1.9	11.4	21.3
BOIN	35.1	21.0			28.0
TITE- BOIN	24.7	21.0			21.6
EI-TITE- BOIN	25.2	20.4	2.7	13.6	21.0
Fixed Scenario 6					
mTPI	27.3	21.0			28.0
TITE-mTPI	16.2	21.0			22.8
EI-TITE-mTPI	16.9	18.4	12.3	47.7	20.4
Keyboard	29.0	21.0			28.0
TITE- Keyboard	16.9	21.0			23.2
EI-TITE- Keyboard	17.3	20.7	1.2	9.0	22.9
BOIN	29.8	21.0			28.0
TITE- BOIN	16.5	21.0			23.1

Design	%MTD	Ave Pts	%reduc Pts	%early comp	Duration
EI-TITE- BOIN	16.1	20.7	1.6	10.2	22.7
Random Scenario 1 ($\mu = 0.5$)					
mTPI	42.7	21.0			28.0
TITE-mTPI	41.0	21.0			19.5
EI-TITE-mTPI	38.6	15.3	27.0	65.7	14.7
Keyboard	44.2	21.0			28.0
TITE- Keyboard	42.1	21.0			19.8
EI-TITE- Keyboard	41.7	19.2	8.8	33.8	17.9
BOIN	45.1	21.0			28.0
TITE- BOIN	40.6	21.0			19.7
EI-TITE- BOIN	41.1	18.6	11.5	37.1	17.4
Random Scenario 2 ($\mu = 1.0$)					
mTPI	73.1	21.0			28.0
TITE-mTPI	72.5	21.0			21.3
EI-TITE-mTPI	68.8	16.4	21.9	61.9	17.2
Keyboard	73.9	21.0			28.0
TITE- Keyboard	71.0	21.0			21.3
EI-TITE- Keyboard	71.6	19.2	8.5	34.7	19.5
BOIN	74.1	21.0			28.0
TITE- BOIN	71.4	21.0			21.3
EI-TITE- BOIN	71.6	18.8	10.4	37.7	19.1

Supplemental Table 7. Detailed Dose escalation and de-escalation boundaries (TTL=0.3)

Design	Action	Num of patients treated at current dose								
		1	2	3	4	5	6	7	8	9
mTPI	Escalate $\leq$	0	0	0	0	0	1	1	1	1

	De-escalate ≥	1	1	2	2	2	3	3	4	4
Keyboard	Escalate ≤	0	0	0	0	1	1	1	1	2
	De-escalate ≥	1	1	2	2	2	3	3	3	4
BOIN	Escalate ≤	0	0	0	0	1	1	1	1	2
	De-escalate ≥	1	1	2	2	2	3	3	3	4

Design	Action	Num of patients treated at current dose								
		10	11	12	13	14	15	16	17	18
mTPI	Escalate ≤	1	2	2	2	2	2	3	3	3
	De-escalate ≥	5	5	5	6	6	7	7	8	8
Keyboard	Escalate ≤	2	2	2	3	3	3	3	4	4
	De-escalate ≥	4	4	5	5	5	6	6	6	7
BOIN	Escalate ≤	2	2	2	3	3	3	3	4	4
	De-escalate ≥	4	4	5	5	6	6	6	7	7

Supplemental Table 8. Percentage of the MTD selection for EI designs only when the MTD is identified early

Design		Dose Level						%early ident
		1	2	3	4	5	6	
Scenario 1	Pr Tox	0.13	**0.28**	0.41	0.50	0.60	0.70	

Design		Dose Level						%early ident
		1	2	3	4	5	6	
mTPI	%MTD	16.1	**59.2**	21.6	3.0	0.2	0.0	
TITE-mTPI	%MTD	13.5	**60.6**	21.9	3.7	0.3	0.0	
EI-TITE-mTPI	%MTD	24.2	**48.7**	22.6	4.2	0.3	0.0	98.0
Keyboard	%MTD	14.5	**57.1**	23.9	4.2	0.3	0.0	
TITE- Keyboard	%MTD	12.8	**58.0**	24.4	4.3	0.5	0.0	
EI-TITE- Keyboard	%MTD	17.7	**59.3**	19.9	2.8	0.2	0.0	78.9
BOIN	%MTD	15.2	**56.4**	24.2	3.9	0.4	0.0	
TITE- BOIN	%MTD	13.2	**59.1**	23.2	4.1	0.4	0.0	
EI-TITE- BOIN	%MTD	18.2	**57.0**	21.3	3.1	0.3	0.0	81.4
Scenario 2	Pr Tox	0.08	0.15	**0.29**	0.43	0.50	0.57	
mTPI	%MTD	1.4	22.8	**55.8**	17.2	2.6	0.3	
TITE-mTPI	%MTD	0.9	21.3	**55.8**	19.0	2.8	0.2	
EI-TITE-mTPI	%MTD	8.1	28.1	**43.1**	17.6	2.7	0.3	95.3
Keyboard	%MTD	0.9	20.4	**56.1**	18.6	3.6	0.4	
TITE- Keyboard	%MTD	0.9	20.1	**56.1**	19.0	3.6	0.3	
EI-TITE- Keyboard	%MTD	2.9	27.3	**51.5**	15.8	2.0	0.5	64.2
BOIN	%MTD	1.0	21.0	**54.8**	19.2	3.7	0.3	
TITE- BOIN	%MTD	1.0	19.9	**56.6**	19.1	3.2	0.3	
EI-TITE- BOIN	%MTD	2.8	25.8	**52.7**	16.1	2.1	0.4	69.9
Scenario 3	Pr Tox	**0.28**	0.42	0.49	0.61	0.76	0.87	
mTPI	%MTD	**78.2**	19.1	2.6	0.1	0.0	0.0	
TITE-mTPI	%MTD	**75.0**	21.9	2.9	0.2	0.0	0.0	
EI-TITE-mTPI	%MTD	**67.9**	26.9	4.8	0.4	0.0	0.0	99.5
Keyboard	%MTD	**73.8**	22.6	3.4	0.2	0.0	0.0	
TITE- Keyboard	%MTD	**72.4**	23.5	3.8	0.3	0.0	0.0	
EI-TITE- Keyboard	%MTD	**73.7**	22.6	3.5	0.3	0.0	0.0	90.8
BOIN	%MTD	**74.0**	22.4	3.4	0.2	0.0	0.0	
TITE- BOIN	%MTD	**73.0**	22.9	3.8	0.3	0.0	0.0	
EI-TITE- BOIN	%MTD	**73.4**	22.6	3.7	0.2	0.0	0.0	92.5
Scenario 4	Pr Tox	0.05	0.10	0.20	**0.31**	0.50	0.70	

Design		Dose Level						%early ident
		1	2	3	4	5	6	
mTPI	%MTD	0.2	5.0	36.7	**48.8**	9.1	0.2	
TITE-mTPI	%MTD	0.2	4.5	35.7	**48.7**	10.7	0.3	
EI-TITE-mTPI	%MTD	3.4	14.7	35.4	**36.5**	9.7	0.3	91.6
Keyboard	%MTD	0.2	4.4	32.9	**50.2**	12.0	0.4	
TITE- Keyboard	%MTD	0.1	3.7	33.9	**49.7**	12.1	0.5	
EI-TITE- Keyboard	%MTD	0.5	8.4	38.1	**43.9**	8.6	0.4	56.9
BOIN	%MTD	0.2	4.3	32.5	**50.9**	11.9	0.3	
TITE- BOIN	%MTD	0.1	4.3	34.5	**49.6**	11.3	0.3	
EI-TITE- BOIN	%MTD	0.4	8.4	38.9	**43.6**	8.2	0.5	62.3
Scenario 5	Pr Tox	0.06	0.08	0.12	0.18	**0.30**	0.41	
mTPI	%MTD	0.2	1.2	7.7	31.4	**41.9**	17.6	
TITE-mTPI	%MTD	0.1	1.7	9.3	32.1	**40.2**	16.6	
EI-TITE-mTPI	%MTD	5.0	8.3	15.9	28.9	**28.4**	13.6	88.4
EI-TITE-mTPI*	%MTD	2.3	7.2	18.4	35.7	**36.1**	0.3	47.6
Keyboard	%MTD	0.1	0.6	5.4	27.8	**44.9**	21.2	
TITE- Keyboard	%MTD	0.1	1.1	7.0	30.5	**43.6**	17.8	
EI-TITE- Keyboard	%MTD	0.8	3.0	10.5	26.6	**32.3**	26.9	51.2
EI-TITE- Keyboard*	%MTD	0.5	2.9	15.8	37.9	**41.2**	1.8	15.2
BOIN	%MTD	0.1	0.8	5.8	27.3	**44.9**	21.1	
TITE- BOIN	%MTD	0.1	1.0	7.7	31.7	**42.5**	17.1	
EI-TITE- BOIN	%MTD	0.6	2.7	11.8	31.3	**30.6**	23.0	55.3
EI-TITE- BOIN*	%MTD	0.6	3.6	18.3	40.5	**35.9**	1.2	19.9
Scenario 6	Pr Tox	0.05	0.06	0.08	0.11	0.19	**0.32**	
mTPI	%MTD	0.1	0.2	1.5	9.4	36.6	**52.1**	
TITE-mTPI	%MTD	0.1	0.5	2.7	11.2	37.9	**47.6**	
EI-TITE-mTPI	%MTD	3.3	4.8	7.7	14.0	28.6	**41.5**	88.6
Keyboard	%MTD	0.0	0.2	1.0	6.7	34.7	**57.4**	
TITE- Keyboard	%MTD	0.0	0.3	1.5	9.7	38.2	**50.2**	
EI-TITE- Keyboard	%MTD	0.3	1.2	2.9	7.5	25.4	**62.8**	62.0
BOIN	%MTD	0.0	0.2	0.9	6.4	34.5	**58.0**	

Design		Dose Level						%early ident
		1	2	3	4	5	6	
TITE- BOIN	%MTD	0.0	0.4	2.0	10.5	38.1	**49.0**	
EI-TITE- BOIN	%MTD	0.4	1.0	3.2	9.1	25.5	**60.8**	62.3

*: The early identification was conducted under a threshold of 0.5.

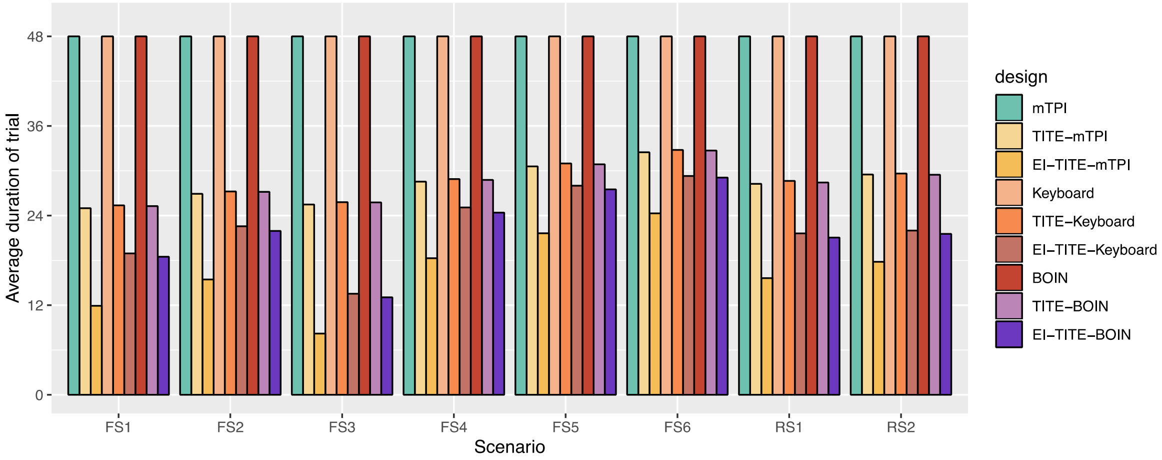

Supplemental Figure 1. Average observed duration

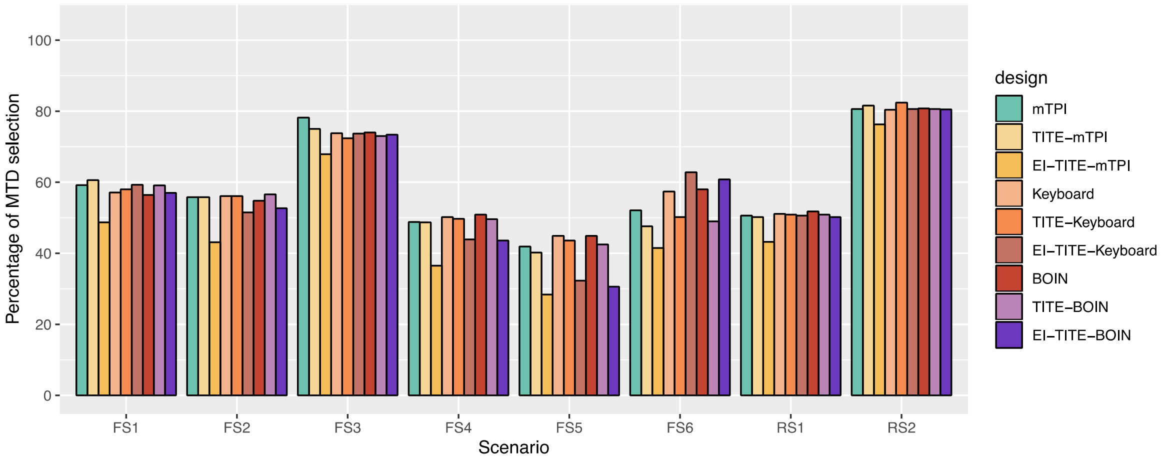

Supplemental Figure 2. Percentage of the correct MTD selection for EI designs only when the MTD is identified early.
